# Comparative evaluation of obturation techniques for C-shaped canals: a network meta-analysis

**DOI:** 10.3389/fdmed.2026.1903167

**Published:** 2026-08-12

**Authors:** Yaojia Lu, Lingjiao Lu

**Affiliations:** Department of Stomatology, The Second Affiliated Hospital of Jiaxing University, Jiaxing, Zhejiang, China

**Keywords:** Continuous Wave Obturation System, C-shaped root canals, network meta-analysis, root canal obturation, root filling technique, single cone technique

## Abstract

**Objective:**

To compare the filling quality of different root canal filling methods in C-shaped canals using a network meta-analysis.

**Materials and methods:**

This PROSPERO-registered study [CRD420251044833] included *in vitro* investigations on C-shaped canals. After screening 1139 records, 9 studies were eligible. A Bayesian model network meta-analysis was performed using R software, with interventions ranked by SUCRA values. Risk of bias was assessed using the RoBDEMAT tool, and subgroup analyses were conducted for different canal configurations and imaging methods.

**Results:**

No statistically significant differences were found between LC and any of the other filling method, with the exception that UC exhibited significantly lower porosity than LC at the middle region(MD = −1.7,95% Crl:-3.3, −0.062). Subgroup analyses revealed that the overall results were modified to some extent by differences in C-shaped canal anatomy and the method used to detect obturation porosity.

**Conclusions:**

Current obturation techniques remain unable to achieve completely void-free filling in C-shaped root canals. However, the obturation performance of the same technique varied across different root levels, suggesting a site-dependent pattern of filling quality.

**Clinical relevance:**

The present findings do not support a definitive recommendation for any particular obturation technique in clinical practice. Rather, they may be viewed as an evidence map that provides a reference framework for the design of future studies.

**Systematic Review Registration:**

https://www.crd.york.ac.uk/prospero, CRD420251044833.

## 1 Introduction

The C-shaped root canal system is an anatomical characterized by its C-shaped cross-sectional morphology (1). They are most frequently observed in mandibular second molars (2), but have also been reported in mandibular first premolars (3), maxillary first molars, and mandibular third molars (4). The formation of the C-shaped root canal has been attributed to incomplete fusion of the Hertwig's epithelial root sheath on the buccal or lingual surface of the root. Additionally, root fusion resulting from the deposition of cementum over time may contribute to the formation of the C-shaped canal (5). The key anatomical feature is the presence of isthmuses connecting root canals, creating complex three-dimensional configurations that vary along the root length (6), which complicates thorough cleaning and complete filling.

In 1991, Melton et al. proposed a classification system for C-shaped root canals based on cross-sectional morphology (7). This classification was subsequently refined by Fan et al. in 2004 through incorporation of both anatomical and radiographic characteristics. This classification system comprises five distinct categories: Type I (C1) demonstrates a continuous C-shaped configuration without any separation; Type II (C2) exhibits a semicolon-like morphology with an interruption of ≥60° in the C-shaped outline; Type III (C3) contains two or three discrete canals with separation angles of <60°; Type IV (C4) presents an isolated round or oval canal near the apex; and Type V (C5) shows no discernible canal orifice, a finding typically observed in apical regions (2).

The goal of root canal obturation is to achieve three-dimensional hermetic sealing of the root canal system, thereby preventing bacterial contamination and reinfection while creating favorable conditions for the healing of periapical tissues (8). Consequently, the selection of an appropriate obturation technique is clinically paramount. Current clinical options include Cold lateral compaction technique (LC), Core Carrier System (CC), Continuous Wave Obturation System (CW), Single cone technique(SC) and so on. However, to date, comparisons of these techniques have primarily focused on conventional root canal systems, with limited evidence available for C-shaped canal configurations. Therefore, this study aims to systematically evaluate and compare the obturation quality of different techniques in C-shaped canals through comprehensive assessment, ultimately identifying the most suitable obturation strategy for these anatomically complex cases.

## 2 Method

This study was conducted in accordance with the Preferred Reporting Items for Systematic Reviews and Meta-Analyses for Network Meta-Analyses (PRISMA-NMA) guidelines (the checklist can be found in Supplementary Appendix 1) and has been registered in the PROSPERO database (https://www.crd.york.ac.uk/prospero), with registration number CRD420251044833.

### 2.1 Search strategy and eligibility criteria

Electronic databases, including PubMed, Web of Science, and Embase, were systematically searched. The search period covered from the inception of the databases up until 30 June 2026. A combination of subject headings and free text terms was used for the search, supplemented by manual searches to ensure comprehensive coverage. The search strategy for PubMed is provided as an example in Table 1. Additionally, grey literature was retrieved from ProQuest and MedRxiv. A detailed description of the search strategy can be found in Supplementary Appendix 2.

**Table 1:** Search strategy of PubMed.

Database	Search strategy
PubMed	(((((((((((((((((((((((“Root Canal Obturation"[Mesh]) OR (Canal Obturation, Root)) OR (Canal Obturations, Root)) OR (Obturation, Root Canal)) OR (Obturations, Root Canal)) OR (Root Canal Obturations)) OR (Endodontic Obturation)) OR (Endodontic Obturations)) OR (Obturation, Endodontic)) OR (Obturations, Endodontic)) OR (Obturation Technique)) OR (Root Canal Filling)) OR (Root Canal Fillings)) OR (sealer-based Obturation)) OR (“Gutta-Percha"[Mesh])) OR (warm vertical compaction)) OR (thermoplasticized obturation)) OR (carrier-based obturation)) OR (voids)) OR (porosity)) OR (micro-CT)) OR (microcomputed tomography)) AND (((((((c shape root canal) OR (c shaped root canal)) OR (c shape root canals)) OR (c shaped root canals)) OR (C-shaped canal system)) OR (C-shaped molar)) OR (C-shaped mandibular molar))) AND ((“1966/01/01"[Date - Publication]: “2026/06/30"[Date - Publication]))

#### 2.1.1 Inclusion and exclusion criteria

The inclusion criteria for studies followed the PICOS framework:

P (Population): Permanent molar with C-shaped root canal systems
I (Intervention): Different root canal filling techniques applied to the experimental teeth
C (Comparison): Studies with a control group
(Outcome): The porosity of root canal filling
S (Study design): in vitro studies

#### 2.1.2 Exclusion criteria

1. Non-C-shaped root canal systems
2. Primary teeth or young permanent teeth
3. Clinical studies, animal studies, case reports, or conference abstracts

### 2.2 Study selection and data extraction

Two independent reviewers screened the literature and extracted the data based on the inclusion and exclusion criteria. Any discrepancies between the reviewers were resolved through discussion. The data extracted included the basic study information (title, first author, year of publication), sample type, sample size, root canal filling methods, and outcome measures.

### 2.3 Quality assessment

The risk of bias for the included studies was assessed using the RoBDEMAT tool (9). The assessment included the following domains: D1 – bias in planning and allocation; D2 – bias in sample/specimen preparation; D3 – bias in outcome assessment; and D4 – bias in data treatment and outcome reporting. A detailed description of the items and evaluation criteria is provided in Supplementary Appendix 3. Each item was rated as “sufficiently reported,” “insufficiently reported,” “not reported,” or “not applicable.” A rating of “sufficiently reported” was considered low risk, “insufficiently reported” was considered moderate risk, “not reported” was considered high risk, and “not applicable” was used when the item was deemed irrelevant or inappropriate for the study. The quality assessment was performed independently by two reviewers, and disagreements were resolved through discussion. If consensus could not be reached, a third reviewer was consulted.

### 2.4 Meta-analysis

Root canal filling porosity, a continuous variable, was used as the primary outcome. Subgroup analysis was conducted based on different C-shaped root canal classifications and the imaging technique employed. Mean differences (MD) and their corresponding 95% confidence intervals (CI) were used as the effect size. A Bayesian model was used for the network meta-analysis. It was conducted using the *gemtc* package in R (version 4.4.3) to generate network plots, cumulative ranking probability curves (Surface Under the Cumulative Ranking Curve, SUCRA), and forest plots. Heatmaps were created using Excel. Sensitivity analysis was performed using the Leave One Out method. The consistency between direct and indirect evidence was assessed using the node-splitting method. The effectiveness of each intervention was ranked according to the estimated probability (SUCRA) and effect size, with smaller values indicating higher rankings. The significance level was set at *α* = 0.05. Given the potential differences among the included studies, clinical and methodological heterogeneity across subgroups was expected beforehand. We used the splitting method for subgroup analysis, and a random-effects model was preferentially adopted for the subgroup analyses. To further ensure the statistical validity of this model choice, we also used a fixed-effects model. We assessed both the I^2^ statistic for heterogeneity quantification and the deviance information criterion (DIC) for model fit assessment. Specifically, when the I^2^ value within a subgroup was <50% and the DIC of the random-effects model did not increase substantially compared with that of the fixed-effect model (*Δ*DIC < 5), the random-effects model was retained as a conservative choice. When the random-effects model yielded a lower DIC with *Δ*DIC > 5, it was directly selected as the superior model. For subgroups comprising two or fewer studies, only descriptive analysis was performed.

## 3 Results

### 3.1 Search results

**Figure 1:**
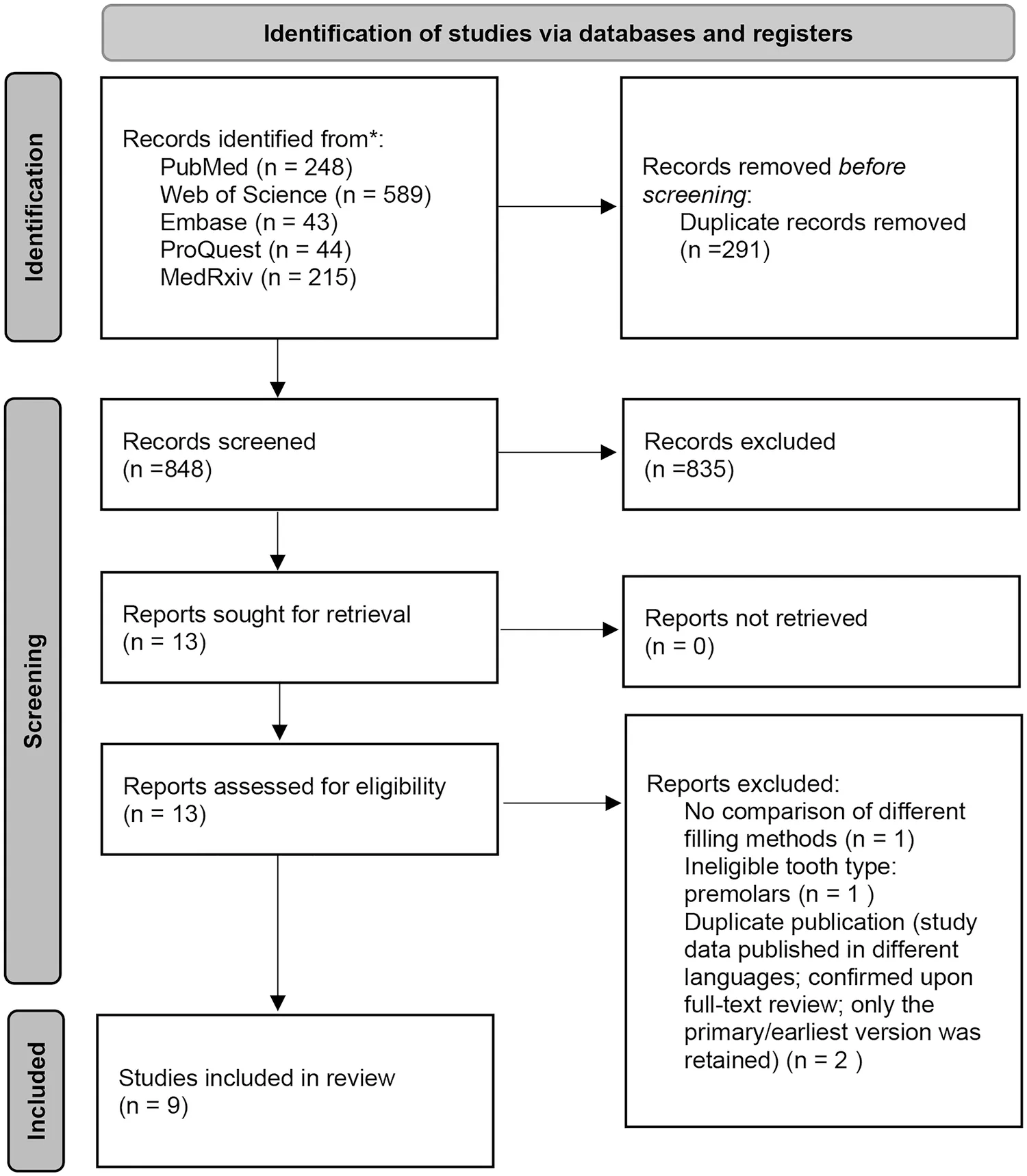
Flow diagram of study selection (based on PRISMA 2020: http://prisma-statement.org/).

Through electronic and manual searches, 1139relevant articles were identified. After removing duplicates, 848articles remained. Following title and abstract screening, 13 articles were selected for full-text review. Of these, 1 article was excluded for studying only a single root canal filling technique, and another was excluded because it used premolars instead of permanent molars, which deviated from our inclusion criteria. Additionally, During full-text screening, two articles were excluded due to duplicate publication—they were identified as reporting the same study data in different languages, and only the most comprehensive report was retained for analysis. A total of 9 *in vitro* studies were included (10–18). The literature screening process, based on PRISMA 2020, is depicted in Figure 1.

### 3.2 Descriptive analysis

This review included 9 studies (10–18), comprising 424 teeth with C-shaped root canals, published between 2015 and 2026. Most studies used 3D-printed tooth models (10–14, 16, 17), with only two study using extracted teeth (15, 18). The study by Gok T (10) included both C2 and C3 root canal types, while Karatekin AO (11) compared C1 and C2 root canal types. The study by Fu et al. (16) utilized teeth that simultaneously exhibited a C2 configuration in the middle and coronal thirds and a C3 configuration in the apical third. Wisawawatin, D (15) selected extracted teeth with C1 and C3 root canal types and randomized them into groups. Bae,G et al. (18), despite also using extracted teeth, did not specify the C-shaped canal configuration of the teeth selected for their experiments. The remaining four studies employed 3D-printed models with C1 root canal types. All studies stored the teeth at 37°C for one week after root filling, followed by imaging and analysis. Seven different root canal filling techniques were used across the studies: Cold Lateral Compaction (LC) technique, Core Carrier System (CC), Continuous Wave Obturation System (CW), Injectable Cold Filling Method (ICF), Single Cone technique with hydraulic calcium silicate-based root canal sealer (SC), Ultrasonic Compaction (UC), and Cold Hydraulic Compaction (CHC). The basic characteristics of the included studies are summarized in Table 2.

**Table 2:** Characteristics of 9 included studies in the network meta-analysis.

Study, year	Sample size	Specimen categories	Obturation technique	Outcome measures
Gok T，2025 (10)	80	3D-printed teeth	LC CC CW SC	voids percentages in apical, middle, coronal
Karatekin AO，2019 (11)	20	3D-printed teeth	LC CW	gutta-percha, sealer and voids percentages in apical, middle, coronal
Soo WKM，2015 (12)	40	3D-printed teeth	LC UC CW CC	gutta-percha, sealer and voids percentages in apical, middle, coronal
Gok T，2017 (13)	80	3D-printed teeth	CC CW LC ICF	gutta-percha, sealer and voids percentages in apical, middle, coronal
Gharechahi M，2024 (14)	60	3D-printed teeth	SC UC CHC	sealer and voids percentages in apical, middle, coronal
Wisawawatin D，2023 (15)	36	Extracted teeth	CHC CW LC	voids percentages in apical, middle, coronal
Fu, D,2025 (16)	32	3D-printed teeth	CW SC	voids percentages in apical, coronal
Gharechahi, M,2026 (17)	20	3D-printed teeth	CW LC	gutta-percha, sealer and voids percentages in apical, middle, coronal
Bae, G,2026 (18)	30	Extracted teeth	CW SC UC	voids percentages in apical, middle, coronal

### 3.3 Risk of bias

**Figure 2:**
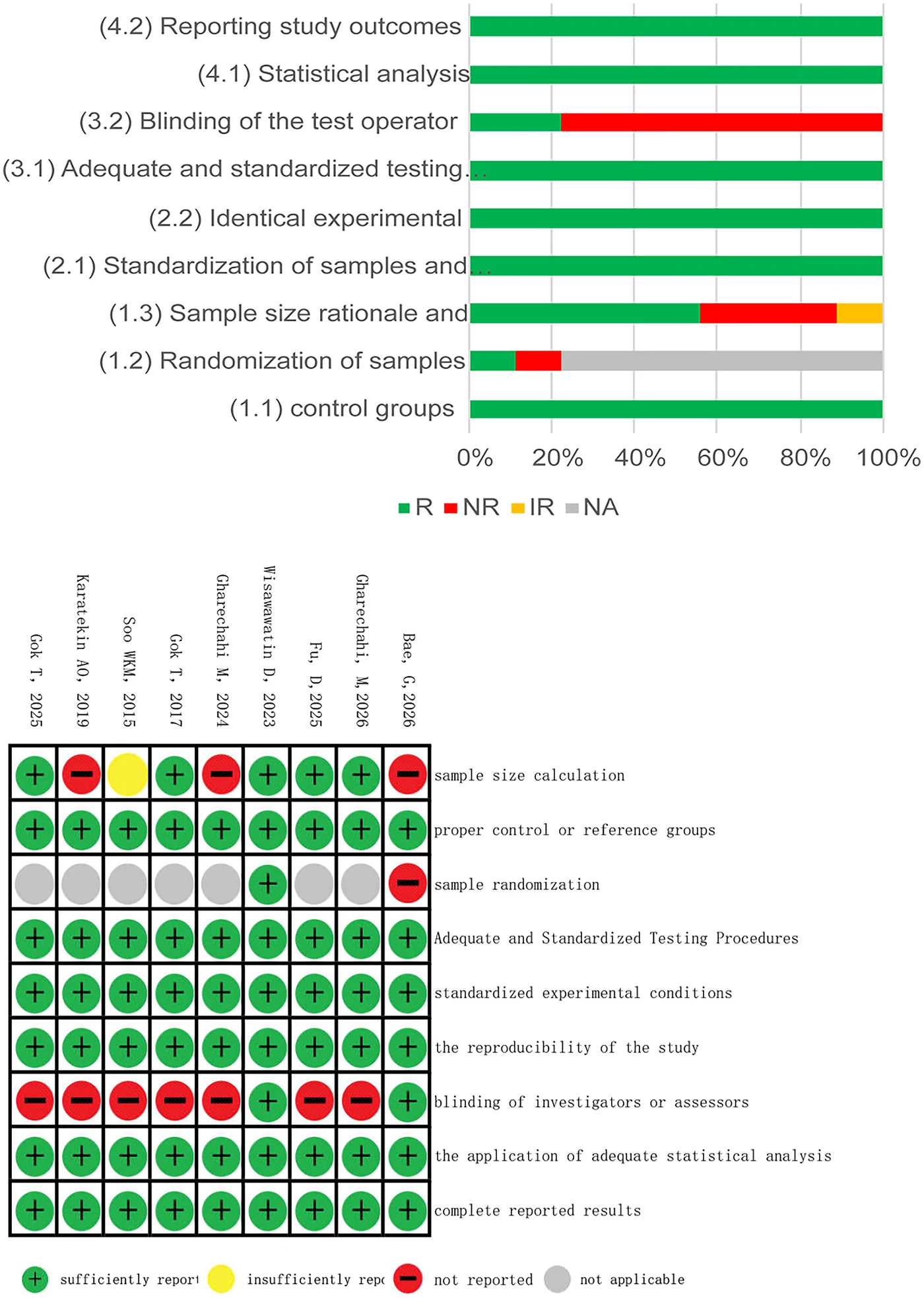
Authors’ judgments about each risk of bias item for each included *in vitro* study, classified as sufficiently reported, insufficiently reported, not reported and not applicable.

The risk of bias for the included studies is shown in Supplementary Appendix 4 and Figure 2. All studies had standardized sample preparation protocols and imaging collection methods, performed reasonable data analysis, and reported complete outcome data. Five studies (10, 13, 15–17) conducted appropriate sample size calculations, while the remaining four did not mention the sample size calculation method. The study by Wisawawatin D (15) randomized the extracted teeth and implemented blinding of the assessor. Bae et al. applied blinding to the outcome assessors in their study; however, no information was provided regarding the allocation method for the included extracted teeth (18). Apart from the investigations by Bae et al. (18) and Wisawawatin et al. (15), blinding during outcome assessment was not reported in any of the remaining seven articles, which represents a notable aspect of the risk of bias in the studies included in the present review. Considering that blinding of the outcome assessor, sample size calculation, and randomization of extracted teeth are several important sources of bias, the present study was classified as moderate risk.

### 3.4 Meta-analyses

#### 3.4.1 Primary outcomes

**Figure 3:**
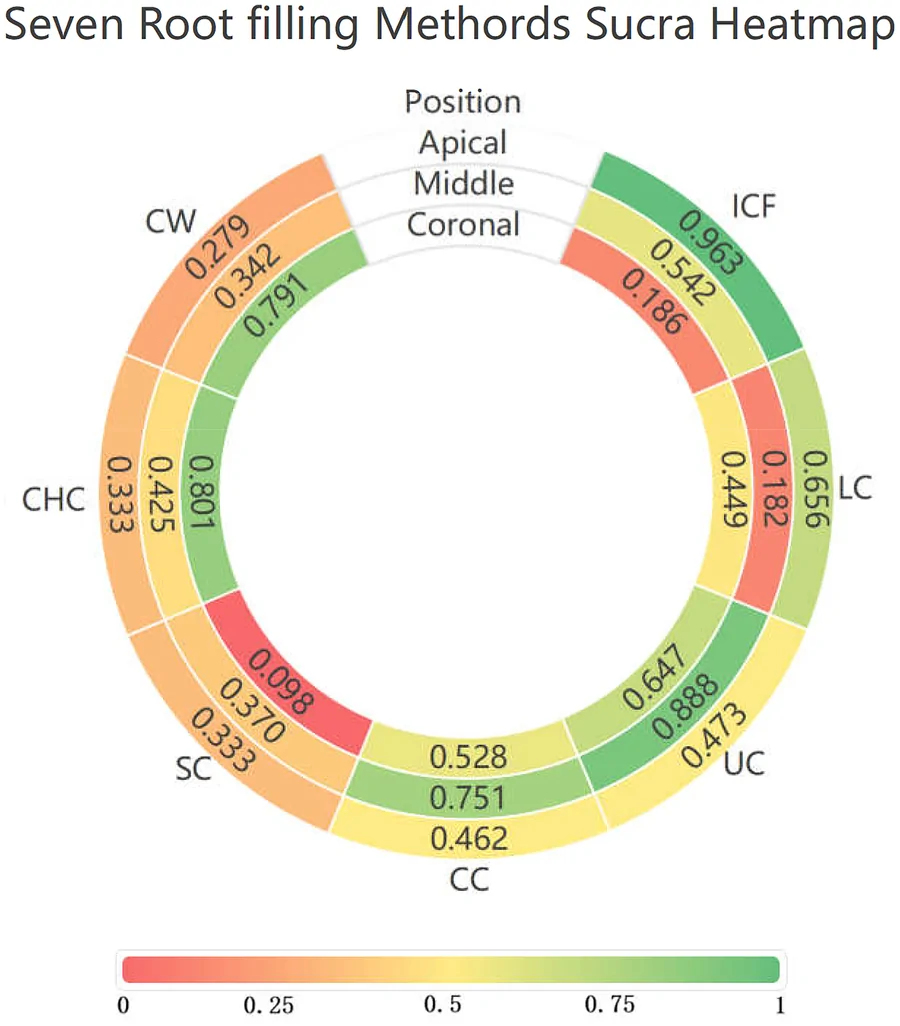
Heatmap of treatment comparisons.

The studies were categorized into three groups for the network meta-analysis (NMA) based on root canal filling location: apex, middle, and coronal regions. A Bayesian model (random effects model) was used for the network meta-analysis, and inconsistency was tested using the node-splitting method, with comparisons between all groups showing *P* > 0.05. The forest plots for node-splitting in the apical, middle, and coronal regions can be found in Supplementary Appendix 5. These findings confirmed statistical consistency between direct and indirect comparisons, supporting the use of a consistency model. Figure 3 presents a heatmap summarizing the SUCRA values of different root filling techniques by region. In this heatmap, the inner layer represents the coronal region, followed by the middle and apical regions in succession. Darker red indicates lower SUCRA values, meaning higher porosity, while darkergreen indicates lower porosity.

**Figure 4:**
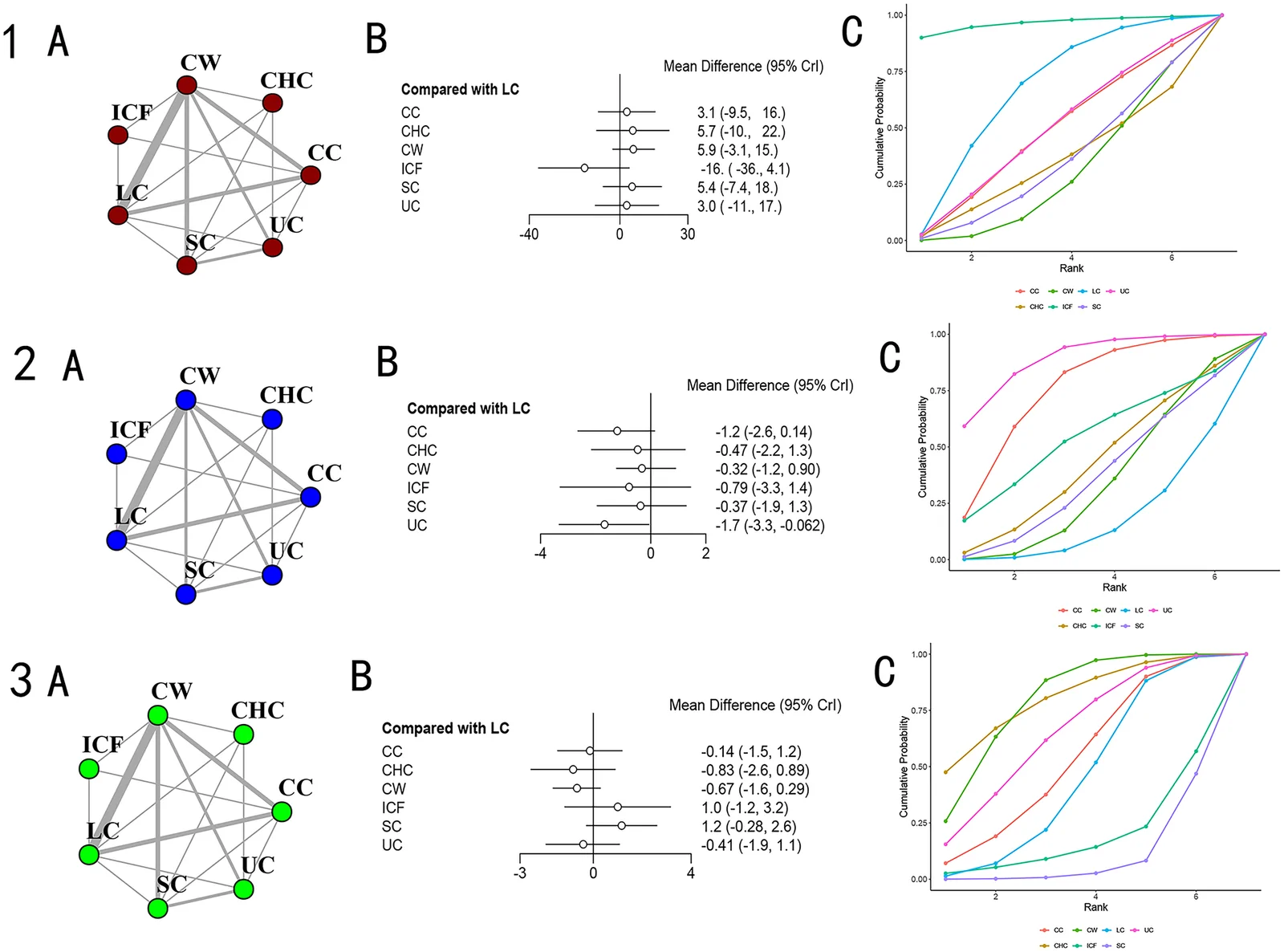
Network meta-analysis (NMA) of void percentages. **(A)** Network diagrams of compared groups, **(B)** Forest plots showing mean differences (95% CrI) versus LC, and **(C)** SUCRA curves for probabilistic ranking.

 Figure 4 shows the results of the network meta-analysis for root canal filling porosity. Panel 4-1 displays the results for the apical region, Panel 4-2 shows the middle region, and Panel 4-3 illustrates the coronal region. Panels 4-1A, 4-2A, and 4-3A show the network evidence plots. These results indicate that in all three NMA groups, both LC and CW were directly compared with all other arms. Additionally, the greatest number of direct comparisons occurred between LC and CW. Panels 4-1B, 4-2B, and 4-3B show the forest plots comparing each filling method to LC in the apical, middle, and coronal regions, respectively. The results showed that, except for the middle region, which was significantly lower in the UC group than in the LC group(MD = −1.7,95% Crl:-3.3，-0.062), no statistically significant differences were found between LC and any of the other filling method at any of the three root levels (apical, middle, and coronal). However, the cumulative probability curves (Panels 4-1C, 4-2C, and 4-3C) suggest differences between filling methods. In the apical region, Panel 4-1C shows that ICF had the lowest filling porosity, ranking first in cumulative probability (SUCRA = 96.27%), indicating its superior filling performance. The ranking, from highest to lowest SUCRA value, is as follows: ICF (SUCRA = 96.27%) > LC (SUCRA = 65.58%) > UC (SUCRA = 47.33%) > CC (SUCRA = 46.23%) > SC (SUCRA = 33.33%) > CHC (SUCRA = 33.31%) > CW (SUCRA = 27.93%). In the middle region, Panel 4-2C shows that UC had the lowest porosity (SUCRA =88.76%), followed by CC (SUCRA =75.13%), while the highest porosity was seen in the LC group (SUCRA = 18.21%). In the coronal region (Panel 4-3C), the CHC group had the lowest filling porosity (SUCRA = 80.08%), followed by CW (SUCRA = 79.08%), while SC showed the worst performance (SUCRA = 9.8%).

#### 3.4.2 Sensitivity analysis

**Figure 5:**
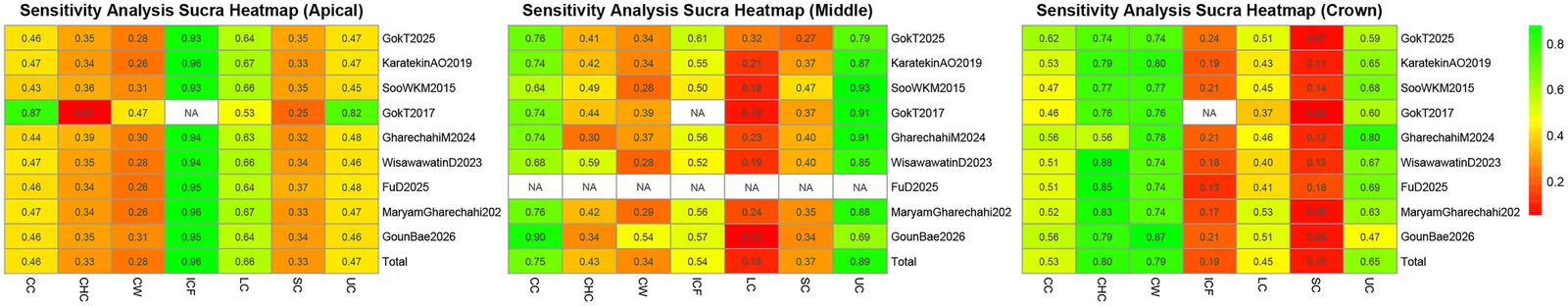
Sensitivity analysis heatmap of SUCRA value. Rows show both experimental groups (bottom) and root canal regions (apical, middle, coronal; top). Columns represent individual studies excluded in sequence. Color gradient indicates ranking performance, with red denoting low SUCRA values (inferior ranking) and green indicating high values (superior ranking).

 Figure 5 presents a heatmap summarizing the results of the sensitivity analysis using the leave-one-out method. Darker red colors indicate smaller SUCRA values, reflecting poorer filling outcomes. The right vertical axisshows the studies excluded during each iteration. From the changes in values and color, it is apparent that excluding the study by Gok T (13) resulted in significant changes in the ranking outcome for the apical region. This suggests that the results are influenced by this particular study, which may undermine the statistical robustness of the findings and limit their generalizability.

#### 3.4.3 Subgroup analysis

For the subgroup analyses, we were unable to perform classical regression-based approaches due to the limited total number of included studies (*n* = 9), and therefore adopted the splitting method. The I^2^ and DIC values for each subgroup are detailed in the Supplementary Appendix 6. For the subgroup analysis based on C-shaped canal typing, the included studies were categorized into three subgroups: C1, C2, and C3. The C1 group comprised seven studies, in which a random-effects model was employed for network meta-analysis, and the SUCRA ranking order is presented in Table 3. The results indicated that, at the apical level, the ranking of interventions was largely in agreement with that observed in the overall analysis. Specifically, ICF, LC, and UC remained the top three ranked interventions (in that order), whereas CW ranked last. At the middle third, the interventions were ranked in descending order of SUCRA values as follows: CC, ICF, CW, UC, CHC, LC, and SC. This ranking represented a marked shift from the overall analysis. At the coronal third, a rank reversal was observed between CW and CHC when comparing the overall analysis with the C1 subgroup. Nevertheless, given the close proximity of their SUCRA values and their stable occupancy of the top two ranks, this reversal is unlikely to be of practical significance and can be deemed negligible. Additionally, UC, CC, and LC, which ranked in the middle tier, exhibited positional interchanges, but their SUCRA values were also comparable. For the C3 subgroup, which included only two studies, only descriptive reporting was performed. For the C2 subgroup, although three studies were available, two were single-arm studies and only one was a multi-arm study, resulting in an extremely unstable network; therefore, descriptive reporting was also adopted for this subgroup. Forest plots for the direct comparisons among interventions within the C2 group are presented in Supplementary Appendix 7.

**Table 3:** I^2^, DIC values, and SUCRA rankings for the C1 group.

Position	Groups	I^2^	DIC	Ranking of sucra value
Apical	C1	6%	36.19	ICF > LC > UC > SC > CC > CHC > CW
	Total	6%	54.33	ICF > LC > UC > CC > SC > CHC > CW
Middle	C1	23%	37.67	CC > ICF > CW > UC > CHC > LC > SC
	Total	24%	53.94	UC > CC > ICF > CHC > SC > CW > LC
Coronal	C1	6%	35.88	CW > CHC > CC > LC > UC > ICF > SC
	Total	6%	53.66	CHC > CW > UC > CC > LC > ICF > SC

For the subgroup analysis based on the imaging methods, consistent with the prespecified model selection strategy, the random-effects model was selected for the stereomicroscopy and micro-CT groups, as it outperformed the fixed-effect model in both cases. In the stereomicroscopy group, the results at the apical and coronal levels were similar to those of the overall analysis, whereas a considerable discrepancy was observed at the middle third. Conversely, the micro-CT group exhibited the opposite pattern, with substantial deviations from the overall results at the apical and coronal levels, while the SUCRA-based ranking at the middle third was broadly consistent with that of the overall analysis. The detailed SUCRA ranking order is presented in Table 4, and the corresponding network plots, forest plots, and SUCRA curves are provided in Supplementary Appendix 7.

**Table 4:** I^2^, DIC values, and SUCRA rankings for the stereomicroscopy and micro CT group.

Position	Groups	I^2^	DIC	Ranking of sucra value
Apical	Stereomicroscopy	7%	30.13	ICF > LC > UC > SC > CC > CHC > CW
	Micro CT	11%	23.88	UC > CC > CW > LC > SC > CHC
	Total	6%	54.33	ICF > LC > UC > CC > SC > CHC > CW
Middle	Stereomicroscopy	25%	32.7	CC > ICF > CW > UC > LC > CHC > SC
	Micro CT	1%	18.14	UC > CC > SC > CW > CHC > LC
	Total	24%	53.94	UC > CC > ICF > CHC > SC > CW > LC
Coronal	Stereomicroscopy	7%	29.99	CHC > CW > CC > UC > LC > SC > ICF
	Micro CT	10%	24.12	UC > CW > CHC > LC > CC > SC
	Total	6%	53.66	CHC > CW > UC > CC > LC > ICF > SC

## 4 Discussion

The primary objective of root canal filling is to establish a fluid-tight seal within the root canal system, preventing bacterial infiltration and trapping any remaining microorganisms after cleaning and shaping to avoid persistent or recurrent infections in the canal space (19). The complexity and high variability of C-shaped root canal anatomy make the filling process more challenging. This study utilized network meta-analysis (NMA) to compare the quality of various root canal filling techniques in C-shaped root canals, drawing on data from nine *in vitro* studies. NMA allows for multiple comparisons simultaneously and synthesizes direct and indirect evidence, providing more accurate results compared to traditional meta-analysis, especially when direct evidence from key studies is limited. Porosity in the root canal filling region may harbor bacteria and their byproducts, provide channels for bacterial leakage, compromising the success of the treatment (20–23). Therefore, this study used porosity as an indicator for evaluating the quality of root canal fillings.

Sensitivity analysis using a leave-one-out method revealed some instability in the study's findings. Specifically, after excluding Gok T (13) in the apical region, the rankings of several experimental groups changed significantly. Among the nine included studies, only Gok T (13) used the ICF filling method, which implied that the effect estimate for this treatment was solely dependent on the findings of this single study, thereby increasing the uncertainty of the analysis. Moreover, ICF exhibited the numerically lowest apical porosity in this study, which could lead to an abnormally high SUCRA value. Since no other studies have replicated this treatment method, the reliability of the conclusion regarding the lowest apical porosity for ICF is therefore reduced. Additionally, the root canal preparation methods and root canal sealers used in these studies were not uniform. Although excluding Gok T (13) led to more consistent results, the small number of included studies and the fact that ICF was only used in this study meant that we decided to retain Gok T (13) in our analysis.

This study analyzed the porosity of root canal fillings according to the observation sites. The porosity of root canal fillings is influenced by factors such as the operator's experience, the quality of root canal preparation, the canal's anatomical morphology, filling technique, and the properties of the sealer (24). The results of this study indicate that porosity in root canal fillings is unavoidable. In C-shaped canals, no statistically significant differences were observed among the majority of obturation techniques, with the exception of a significantly lower porosity at the middle third in the UC group compared with the LC control group The ranking and probability estimates derived from the network meta-analysis further suggested that the relative performance of the obturation techniques varied across the different root levels with respect to porosity.

In the apical region, ICF ranked highest, followed by LC, with CW ranked last. LC is the most commonly used reference group, often employing epoxy-based sealers. However, the accessory cones’ inability to fully conform to the irregularities of the canal walls, combined with uneven pressure distribution during the procedure, may result in the formation of gaps (25). ICF uses GuttaFlow Bioseal as the root canal sealer, a premixed, injectable paste containing gutta-percha particles and a silicone-based matrix. This composition combines the sealing ability of solid gutta-percha with the flowability of a paste-type material. Several inherent characteristics account for the effective sealing capacity of GuttaFlow Bioseal: strong dentin adhesion, favorable flow dynamics that allow deep penetration into dentinal tubules, and minimal volumetric expansion as a consequence of its high water absorption capacity (26). These characteristics compensate for the tendency of epoxy-based sealers to experience microleakage. However, it should be noted that only one study in this review used ICF, so the possibility of overestimating its effectiveness cannot be ruled out. In pulp diseases, clinicians are advised to apply a thinner layer of root canal sealer to reduce the risk of microleakage, which may result from sealer shrinkage during setting and long-term dissolution (27). Compared to LC and ICF, other filling methods used fewer gutta-percha points in the apical region, thus requiring more sealer to fill the voids. This may result in an increased proportion of sealer in the apical region, leading to higher porosity. Kim et al. (28) also demonstrated that LC generated significantly less apical porosity compared to SC and CW. Upon heating, the carrier-cone (CC) system softens the gutta-percha, enhancing its adaptability to the canal walls. However, as the filling material enters the root canal, it begins to separate from the carrier, which could potentially increase porosity (29). The apical region of C-shaped root canals is anatomically complex, with a wider apical area. As a result, softened gutta-percha may flow into areas of lesser resistance, such as the isthmus, rather than effectively filling the apical foramen (12). These may contribute to increased porosity in CC fillings. One study has indicated that CW has insufficient heat transfer to the apical region, which directly affects the viscoelasticity of the gutta-percha, significantly reducing its adaptability in the apical area (28). Moreover, using fewer master cones in wider canals may increase the risk of voids. Thus, CC ranked higher in the probabilistic analysis than CW in the apical region. The rankings of UC, SC, and CHC ahead of CW in the middle and coronal regions could be attributed to the use of bioceramic materials, which is known for its superior biocompatibility and sealing performance. The material slightly expands during setting, which can further improve the seal and reduce microleakage (30). Bioceramic materials also release calcium ions, and the hydroxyapatite formed in the calcium silicate hydrate phase strengthens the bond between the dentin wall and the sealer (31). These dimensional stability characteristics may, to some extent, compensate for the susceptibility of epoxy resin-based sealers to microleakage, thereby contributing to a reduction in porosity. Studies have demonstrated that ultrasonic activation reduces the viscosity of sealers, enhancing their flowability and penetration, which allows them to more effectively conform to the complex anatomy of the root canal system. Additionally, ultrasonic activation minimizes the formation of internal voids, thus reducing porosity (32–34). Furthermore, the heat generated by ultrasound can improve the mixing of sealer particles, increasing the cohesive strength between particles (35), thereby reducing the porosity of the root canal filling.

In the middle and coronal regions, both LC and ICF showed lower SUCRA rankings, which may suggest less favorable porosity profiles. A similar trend was also noted in a previous study by Li et al. (36), who confirmed that LC had a significantly higher porosity percentage in oval-shaped root canals. This may be due to insufficient expansion of the middle and coronal portions of the canal, preventing LC from fully filling the accessory cones and achieving adequate lateral compaction, resulting in higher porosity (37). UC and CC exhibited comparable SUCRA values and occupied the top two ranks in the middle region, which may point to a possible advantage for both techniques. The higher ranking observed with UC may still be attributable to the ultrasonic effect described above, which enhances sealer flowability and facilitates the elimination of entrapped air bubbles, thereby creating favorable conditions for complete canal filling. As for CC, its favorable ranking in the probabilistic analysis possibly due to the uniform root canal filling produced by multiple nuclear carrier systems, with a high proportion of gutta-percha-filled areas and a reduced occurrence of voids (36). The higher SUCRA estimates observed for CW in the middle and coronal regions is due to effective heat transfer, which softens the gutta-percha and improves its adaptation to the root canal during vertical compaction (38). This is supported by several studies: Silverio et al. (39) used micro-CT to show that CW achieved a higher gutta-percha ratio in the coronal third of irregular canals compared to LC and CC. Similarly, Kim et al. (28) found that CW produced less porosity in the coronal region compared to SC and LC. Injectable heated gutta-percha formed a more uniform distribution pattern, resulting in fewer sealers and voids (40). These findings suggest that CW may represent a possible mechanism requiring confirmation in the upper sections of the root canal. At the coronal level, CW and CHC ranked among the top two with very close SUCRA values, which may reflect a more favorable trend for both techniques in the probabilistic analysis. The advantage of CHC may be explained by the use of a hydraulic root canal expander, which is inserted before the addition of accessory cones to create space, thereby improving the cold hydraulic sealing quality in C-shaped root canals (41).

We conducted subgroup analysis based on the classification of C-shaped root canals. In the subgroup analysis stratified by C-shaped canal type, the SUCRA-based ranking order in the C1 group at both the apical and coronal levels was generally consistent with that of the overall analysis, suggesting that the overall findings at these two levels are relatively robust. However, at the middle third, the SUCRA-based ranking in the C1 group exhibited a substantial change compared with the overall analysis, primarily manifested as a marked rise in the ranking of CW and a pronounced decline in the rankings of UC and SC. This discrepancy may be attributable to the anatomical peculiarities of C-shaped canals and the technical challenges inherent in their preparation. The different canal configurations in the coronal and middle thirds appear to exert only a limited influence on the quality of apical obturation, which may account for the general consistency between the C1 subgroup and the overall results at the apical level. However, conventional root canal instrumentation techniques are unable to achieve either sufficient coronal flaring or adequate preparation of the isthmus. Consequently, during obturation of the middle third, the gutta-percha points cannot readily adapt to the insufficiently prepared isthmus, leaving the sealer as the primar means of obturating these otherwise inaccessible spaces. During obturation, the vertical compaction forces applied to CW and CC at the coronal aspect may promote sealer flow into the isthmus areas. ICF achieves this effect through lateral condensation. In contrast, neither UC nor SC is subjected to additional compressive forces. Consequently, in the C1 group, where the isthmus is more extensive and anatomically complex (42), CC, CW, and ICF showed a numerically lower porosity, whereas UC and SC showed relatively less favorable outcome. At the coronal level, however, the isthmus tends to be wider and more favorable for obturation, rendering the inherent limitations of C1 type instrumentation and filling less pronounced. This may explain why the C1 group exerted only a marginal influence on the overall results at this level. This suggests that the influence of canal type on obturation quality is unlikely to be uniform across the root; instead, it appears to be site-specific. It's worth noting that this subgroup analysis did not use a classic meta-regression approach. The fluctuation in the ranking of the root-central C1 subgroup, while anatomically reasonable, could also be influenced by the limited number of studies included in this subgroup (which leads to unstable ranking estimates due to insufficient statistical precision).

For the subgroup analysis based on the imaging methods, the SUCRA-based rankings in the stereomicroscopy group were similar to those of the overall analysis at the apical and coronal levels, but diverged considerably at the middle third. Conversely, the micro-CT group showed the opposite pattern. These findings suggest that the imaging modality may serve as an important effect modifier influencing the pooled evidence. Micro-computed tomography (micro-CT) is a three-dimensional nondestructive imaging technique that enables 3D reconstruction and observation from arbitrary angles, with the capacity to detect micron-scale interfacial gaps and sealer penetration. However, it is susceptible to imaging artifacts, which may compromise image quality and analytical accuracy. The major strength of microscopic evaluation is its high lateral resolution, which enables detailed visualization of the material–tissue interface. Nevertheless, its two-dimensional nature restricts the assessment to one or a few selected sections, which may lead to incomplete data and preclude a full understanding of the three-dimensional structure (43). Furthermore, stereomicroscopy relies on staining and manual interpretation of 2D sections, making it prone to observer variability and demanding considerable operator skill. These methodological constraints pose considerable challenges to reproducibility and standardization, which may account for the instability in the subgroup analyses. It should be noted that the limited sample size within subgroups may have amplified the impact of random error on the ranking results. The statistical stability of these findings therefore remains uncertain, and the present subgroup analyses should be interpreted as exploratory. In future research, we recommend either adopting a standardized imaging protocol or employing both methods concurrently to minimize measurement bias.

Notably, with respect to the above-mentioned finding that porosity was significantly lower in the UC group than in the LC group in the middle region, the result reached statistical significance only when either the study by Soo WKM 2015(MD = −2.6, 95% CrI: −5.2 to −0.22) or the study by Gok T 2017(MD = −1.7, 95% CrI: −3.5 to −0.14) was excluded in the leave-one-out sensitivity analysis. In subgroup analyses, a significant difference in porosity between the LC and UC groups was observed only in the micro-CT subgroup (MD = −4.7, 95% CrI: −8.1 to −1.3), while no significant difference was detected in the C1 group or the stereomicroscopy group. These results indicate that the observed reduction in porosity for UC compared to LC in the middle region is statistically unstable, and further randomized controlled trials are required to confirm this effect.

This review provides valuable insights for clinical practice, but it also has several limitations. Foremost among these is the relatively small number of included studies, with some obturation techniques represented by only a single investigation. Given that the network meta-analysis was constructed on the basis of only nine studies, the statistical power of the multiple-treatment comparison may be limited, rendering the SUCRA rankings statistically unstable. This implies that the apparent superiority of the top-ranked interventions over the second- and third-ranked ones, as suggested by the SUCRA values, may not be statistically significant. For certain subgroups, the number of eligible studies was extremely small, precluding any meaningful meta-analysis and limiting the ability to formally evaluate potential effect modifiers. Secondly, all studies included in this review were *in vitro* investigations, which were conducted in controlled environments that do not fully replicate clinical conditions. Variables that may influence the final success rate, such as root canal preparation techniques, irrigation and disinfection protocols, and the experience of clinicians should also be considered. Besides, the structural differences between resin materials and dentin, such as the absence of dentinal tubules and moisture, must be taken into account. Calcium silicate-based sealers, which require moisture for hydration, may be affected by this. The use of 3D-printed root canals could prevent the penetration of environmental moisture, thereby compromising the hydration process and potentially impacting the quality of the filling (44). Therefore, the findings of this study cannot be directly extrapolated to clinical practice. Future research could focus on creating standardized *in vitro* experimental systems, testing methods, and evaluation criteria. Studies with large sample sizes specifically focused on C-shaped root canal systems are essential for a deeper understanding of the performance differences among filling techniques for different C1-C5 subtypes. Additionally, more high-quality clinical studies will help establish evidence-based decision-making trees for selecting root canal filling techniques, providing more precise guidance for clinical practice.

## 5 Conclusion

This study, through a systematic review and network meta-analysis, revealed that current filling techniques are unable to achieve complete gap-free filling in C-shaped root canals. Across most techniques, no statistically significant differences in porosity were observed, with the sole exception that UC exhibited significantly lower porosity than LC at the middle region. Ranking probabilities suggest possible regional differences, but these findings are exploratory and should be interpreted with caution due to the limited number of original studies. The present findings do not support a definitive recommendation for any particular obturation technique in clinical practice. Rather, they may be viewed as an evidence map that provides a reference framework for the design of future studies.

## Data Availability

The original contributions presented in the study are included in the article/Supplementary Material, further inquiries can be directed to the corresponding author.

## Supplementary material

Table 1 Appendix 1 PRISMA NMA Checklist

Table 2 Appendix 3 Sources of bias within each domain and signalling questions

Table 3 Appendix 4 The risk of bias for the included studies

Table 4 Appendix 6 The I² and DIC values for each subgroup

Table 5 Appendix 2 The search strategy

Data Sheet 1 Appendix 5 The forest plots for node-splitting in the apical, middle, and coronal regions

Data Sheet 2 Appendix 7 The subgroup analysis charts (including network plots, forest plots, and SUCRA curve)
