# Supplementary material for: Comparative evaluation of obturation techniques for C-shaped canals: a network meta-analysis
Source: Front Dent Med. 2026 Aug 12;7:1903167. doi: 10.3389/fdmed.2026.1903167 (PMC13508814; doi:10.3389/fdmed.2026.1903167)
Supplement: Supplementary file 2 [file Table2.docx]

Sources of bias within each domain and signalling questions

| Domain | name | Sources of bias | Signalling question(s) | Description |
| --- | --- | --- | --- | --- |
| D1 | Bias in planning and  allocation | (1.1) Control group | Did the study employ one or more control groups (positive or negative or existing standard) in its experimental design? | Control groups are critical to the experimental design. Studies should be assessed for an adequate control group which can be a positive or negative control or an existing standard. Control groups will vary by study topic.  Some studies will require more than one control group and reviewers should use their judgement to classify the presence of control groups as “insufficiently reported” if more control groups are needed. Otherwise, they should be marked as “sufficiently reported” or “not reported”, respectively. |
|  |  | (1.2) Randomization of samples | Was randomization adequately carried out and reported? | Randomization is important in samples whose nature implies intervariability (i.e., teeth). Reviewers should assess whether samples were randomized, and their allocation was concealed appropriately.  Randomization may be conducted using computer generated sequences, random number attribution tables, shuffling envelopes, or cards. Studies that only mention “teeth were randomly allocated” but fail to give details of the randomization process should be marked as “insufficiently reported”.  Otherwise, they should be marked as “sufficiently reported”, when it was adequately carried out, or “not reported”, if not. |
|  |  | (1.3) Sample size rationale and reporting | Did the study provide a rationale and justification for the sample size chosen or feature an a priori power analysis? | Authors are required to justify the rationale for the sample size of their study. This rationale may lie in existing defined standard sample sizes (i.e.  ADM guidelines or ISO standards) or a sample size estimation. Post-hoc power analyses are not recommended, but a priori analyses are highly recommended. In any case, a transparent explanation for the chosen sample size as well as its estimation needs to be provided. To judge this item as “sufficiently reported”, authors should have referred to accepted standards, or should have explained the expected effect size and power level as well as the software used for calculation. The expected effect size should be justified based on a pilot study or previously published studies. Otherwise, studies should be marked as “insufficiently reported” or “not reported”, respectively. |
| D2 | Bias in sample/specimen  preparation | (2.1) Standardization of samples and materials | Were samples and material choice/employment standardized according to the aim of the study? | Reviewers should assess how standardized samples and materials were employed across groups (e.g., different shades of composites being used). If a non-carious teeth study model is adopted, authors should explicitly mention the use and randomization of sound teeth (free of restorations and other defects). Reviewers are expected to judge whether manufacturers’ recommendations were followed for materials (whenever this is applicable to the study design). Studies which involve light-curing (photopolymerisation) of samples must report correct irradiance output, wavelength and tip distance to sample. Reviewers should also assess whether sources and composition of materials are correctly reported. If these details are incomplete or non-existent, they should be marked as “insufficiently reported” or “not reported” |
|  |  | (2.2) Identical experimental conditions across groups | Were the storage, experimental or treatment conditions standardized across samples and materials? | Reviewers should assess whether identical conditions were provided for different experimental groups. Factors such as temperature, humidity, time and equipment settings are expected to be identical and controlled, and indications that suggest otherwise should raise concerns. Authors should specifically indicate storage conditions (solution source, concentration, time) and storage/ageing solution or other reagent preparation methods, to enable reproducibility. Studies that fail to give sufficient information should be marked as “insufficiently reported" or “not reported”. |
| D3 | Bias in outcome  assessment | (3.1) Adequate and standardized testing procedures and outcomes | Were testing procedures and outcome(s) measure(s) explained or defined in sufficient detail to allow reproducibility and critical appraisal? | Reviewers should assess whether the chosen test or test procedure (including equipment or instruments) was adequately described to allow critical appraisal and replication, if needed. Any outcomes and outcome measures should be defined properly to allow interpretation and, if needed, comparison or pooling across studies. Careful consideration should be given to testing procedures being standardized. ADM guidelines or ISO/ASTM standards should be used if applicable and in line with the research question. |
|  |  | (3.2) Blinding of the test operator | Was the test operator blinded to the different experimental groups? | If applicable to the study design, test operators should be blinded to the different experimental groups under testing, and reviewers should appraise this blinding step |
| D4 | Bias in data treatment and  outcome reporting | (4.1) Statistical analysis | Was the statistical analysis adequate and reported in sufficient detail? | Reviewers should assess if the chosen descriptive and analytical statistical approach was adequate to the yielded data and the study aims. This relates to the scale of data (continuous, ordinal, nominal) but also its distribution (e.g. reporting of means or median values or choice of the appropriate statistical test depending on skewness). Specific study design factors (e.g., factorial design, repeated measures at time points) should be appropriately reported and reflected. The software used for statistical evaluation and the applied level of significance should be reported. If relevant information is missing, reviewers should judge whether the study merits “insufficiently reported” or “not reported” |
|  |  | (4.2) Reporting study outcomes | Are all relevant outcome data, expected to be reported, available in sufficient detail? | Reviewers should assess whether the reported outcomes of a study are complete and in line with what could be expected or has been defined as planned outcomes by the researcher before conducting the study, but also if outcomes are reported in sufficient detail for a full appraisal. Reviewers willevaluate if relevant outcome data is missing or incomplete (e.g., are bond strength data reported alongside failure mode data), and if outcome reporting is sufficiently detailed (e.g., measures of precision like confidence intervals or standard errors are expected to be reported if applicable) |
