# Supplementary material for: Comparative evaluation of obturation techniques for C-shaped canals: a network meta-analysis
Source: Front Dent Med. 2026 Aug 12;7:1903167. doi: 10.3389/fdmed.2026.1903167 (PMC13508814; doi:10.3389/fdmed.2026.1903167)
Supplement: Supplementary file 3 [file Table3.docx]

|  | D1. Bias in planning and allocation | | | D2. Bias in sample/specimen preparation | | D3. Bias in outcome assessment | | D4. Bias in data treatment and outcome reporting | |
| --- | --- | --- | --- | --- | --- | --- | --- | --- | --- |
| study | (1.1) control groups | (1.2) Randomization of samples | (1.3) Sample size rationale and  reporting | (2.1) Standardization of samples and materials | (2.2) Identical experimental  conditions across groups | (3.1) Adequate and standardized testing procedures and outcome | (3.2) Blinding of the test operator | (4.1) Statistical analysis | (4.2) Reporting study outcomes |
| Gok T，2025 | R | NA | R | R | R | R | NR | R | R |
| Karatekin AO，2019 | R | NA | NR | R | R | R | NR | R | R |
| Soo WKM，2015 | R | NA | IR | R | R | R | NR | R | R |
| Gok T，2017 | R | NA | R | R | R | R | NR | R | R |
| Gharechahi M，2024 | R | NA | NR | R | R | R | NR | R | R |
| Wisawawatin D，2023 | R | R | R | R | R | R | R | R | R |
| Fu, D,2025 | R | NA | R | R | R | R | NR | R | R |
| Gharechahi, M,2026 | R | NA | R | R | R | R | NR | R | R |
| Bae, G,2026 | R | NR | NR | R | R | R | R | R | R |

Quality analysis of studies included in the systematic review, separated by their risk of bias in different domains. R – sufficiently reported/adequate, NR – not reported; IR – insufficiently reported; and NA – not applicable..

References：

**RoBDEMAT: A risk of bias tool and guideline to support reporting of pre-clinical dental materials research and assessment of systematic reviews**

**Is Cention-N comparable to other direct dental restorative materials? A systematic review with network meta-analysis of in vitro studies**
