# Supplementary material for: Comparative evaluation of obturation techniques for C-shaped canals: a network meta-analysis
Source: Front Dent Med. 2026 Aug 12;7:1903167. doi: 10.3389/fdmed.2026.1903167 (PMC13508814; doi:10.3389/fdmed.2026.1903167)
Supplement: Supplementary file 4 [file Table5.docx]

**PubMed**

((((((((((((((((((((((("Root Canal Obturation"[Mesh]) OR (Canal Obturation, Root)) OR (Canal Obturations, Root)) OR (Obturation, Root Canal)) OR (Obturations, Root Canal)) OR (Root Canal Obturations)) OR (Endodontic Obturation)) OR (Endodontic Obturations)) OR (Obturation, Endodontic)) OR (Obturations, Endodontic)) OR (Obturation Technique)) OR (Root Canal Filling)) OR (Root Canal Fillings)) OR (sealer-based Obturation)) OR ("Gutta-Percha"[Mesh])) OR (warm vertical compaction)) OR (thermoplasticized obturation)) OR (carrier-based obturation)) OR (voids)) OR (porosity)) OR (micro-CT)) OR (microcomputed tomography)) AND (((((((c shape root canal) OR (c shaped root canal)) OR (c shape root canals)) OR (c shaped root canals)) OR (C-shaped canal system)) OR (C-shaped molar)) OR (C-shaped mandibular molar))) AND (("1966/01/01"[Date - Publication] : "2026/06/30"[Date - Publication]))

**Web of science**

#1 ((((((TS=(c shape root canal)) OR TS=(c shape root canals)) OR TS=(c shaped root canal)) OR TS=(c shaped root canals)) OR TS=(C-shaped canal system)) OR TS=(C-shaped molar)) OR TS=(C-shaped mandibular molar)

#2 (((((((((((((((TS=(Root Canal Obturation)) OR TS=(Root Canal Obturations)) OR TS=(Obturation Technique)) OR TS=(Root Canal Filling)) OR TS=(Root Canal Fillings)) OR TS=(Endodontic Obturation)) OR TS=(Endodontic Obturations)) OR TS=(sealer-based Obturation)) OR TS=(gutta-percha)) OR TS=(warm vertical compaction)) OR TS=(thermoplasticized obturation)) OR TS=(carrier-based obturation)) OR TS=(voids)) OR TS=(porosity)) OR TS=(micro-CT)) OR TS=(microcomputed tomography)

#3 DOP=(1966-01-01/2026-06-30)

#4 #1 AND #2 AND #3

Our search was conducted through Zhejiang University’s Web of Science portal, selecting “all Databases” (including: Web of Science Core Collection,BIOSIS Citation Index,BIOSIS Citation Index Database™, Derwent Innovations Index,FSTA® - the food science resource,Grants Index,KCI-Korean Journal Database,MEDLINE®,Preprint Citation Index ProQuest™ Dissertations & Theses Citation Index,Research Commons,SciELO Citation Index, Zoological Record ) for the database, and “all” for the collection (including: Web of Science Core Collection, BIOSIS Citation Index, Chinese Science Citation Database, Derwent Innovations Index, FSTA – the food science resource, Grants Index, KCI-Korean Journal Database, MEDLINE, Preprint Citation Index, ProQuest Dissertations & Theses Citation Index, Research Commons, SciELO Citation Index, and Zoological Record ).

EMBASE

1#'('root canal obturation'/exp OR 'root canal obturation' OR 'gutta percha'/exp OR 'gutta percha') AND [<1966-2026]/py

2#('root canal obturation':ab,ti OR 'root canal

obturations':ab,ti OR 'obturation

technique':ab,ti OR 'root canal filling':ab,ti OR

'root canal fillings':ab,ti OR 'endodontic

obturation':ab,ti OR 'endodontic

obturations':ab,ti OR 'sealer-based

obturation':ab,ti OR 'gutta percha':ab,ti OR

'warm vertical compaction':ab,ti OR

'thermoplasticized obturation':ab,ti OR

'carrier-based obturation':ab,ti OR voids:ab,ti

OR porosity:ab,ti OR 'micro ct':ab,ti OR

'microcomputed tomography':ab,ti) AND [<1966-2026]/py

3# #1or#2

4# ('c shape root canal':ab,ti OR 'c shaped root

canal':ab,ti OR 'c shape root canals':ab,ti OR 'c

shaped root canals':ab,ti OR 'c-shaped canal

system':ab,ti OR 'c-shaped molar':ab,ti OR

'c-shaped mandibular molar':ab,ti) AND [<1966-2026]/py

5# #3 AND #4

**Proquest**

S1：abstract(c shape root canal) OR abstract(c shaped root canal) OR abstract(c shape root canals) OR abstract(c shaped root canals) OR abstract(C-shaped canal system) OR abstract(C-shaped molar) OR abstract(C-shaped mandibular molar)

S2：abstract(Root Canal Obturation) OR abstract(Root Canal Obturations) OR abstract(Obturation Technique) OR abstract(Root Canal Filling) OR abstract(Root Canal Fillings) OR abstract(Endodontic Obturation) OR abstract(Endodontic Obturations) OR abstract(sealer-based Obturation) OR abstract(gutta-percha) OR abstract(warm vertical compaction) OR abstract(thermoplasticized obturation) OR abstract(carrier-based obturation) OR abstract(voids) OR abstract(porosity) OR abstract(micro-CT) OR abstract(microcomputed tomography)

S3：[S1] AND [S4]
